# Brain–Computer Interface Training after Stroke Affects Patterns of Brain–Behavior Relationships in Corticospinal Motor Fibers

**DOI:** 10.3389/fnhum.2016.00457

**Published:** 2016-09-16

**Authors:** Brittany M. Young, Julie M. Stamm, Jie Song, Alexander B. Remsik, Veena A. Nair, Mitchell E. Tyler, Dorothy F. Edwards, Kristin Caldera, Justin A. Sattin, Justin C. Williams, Vivek Prabhakaran

**Affiliations:** ^1^Department of Radiology, University of Wisconsin Hospital and Clinics, University of Wisconsin – Madison, MadisonWI, USA; ^2^Medical Scientist Training Program, University of Wisconsin – Madison, MadisonWI, USA; ^3^Neuroscience Training Program, University of Wisconsin – Madison, MadisonWI, USA; ^4^Department of Biomedical Engineering, University of Wisconsin – Madison, MadisonWI, USA; ^5^Department of Kinesiology and Department of Medicine, University of Wisconsin – Madison, MadisonWI, USA; ^6^Department of Neurology, University of Wisconsin – Madison, MadisonWI, USA; ^7^Department of Orthopedics and Rehabilitation, University of Wisconsin – Madison, MadisonWI, USA; ^8^Department of Neurosurgery, University of Wisconsin – Madison, MadisonWI, USA; ^9^Department of Psychology and Department of Psychiatry, University of Wisconsin – Madison, MadisonWI, USA

**Keywords:** brain–computer interface, stroke, upper extremity, rehabilitation, diffusion tensor imaging, neuroplasticity

## Abstract

**Background:** Brain–computer interface (BCI) devices are being investigated for their application in stroke rehabilitation, but little is known about how structural changes in the motor system relate to behavioral measures with the use of these systems.

**Objective:** This study examined relationships among diffusion tensor imaging (DTI)-derived metrics and with behavioral changes in stroke patients with and without BCI training.

**Methods:** Stroke patients (*n* = 19) with upper extremity motor impairment were assessed using Stroke Impact Scale (SIS), Action Research Arm Test (ARAT), Nine-Hole Peg Test (9-HPT), and DTI scans. Ten subjects completed four assessments over a control period during which no training was administered. Seventeen subjects, including eight who completed the control period, completed four assessments over an experimental period during which subjects received interventional BCI training. Fractional anisotropy (FA) values were extracted from each corticospinal tract (CST) and transcallosal motor fibers for each scan.

**Results:** No significant group by time interactions were identified at the group level in DTI or behavioral measures. During the control period, increases in contralesional CST FA and in asymmetric FA (aFA) correlated with poorer scores on SIS and 9-HPT. During the experimental period (with BCI training), increases in contralesional CST FA were correlated with improvements in 9-HPT while increases in aFA correlated with improvements in ARAT but with worsening 9-HPT performance; changes in transcallosal motor fibers positively correlated with those in the contralesional CST. All correlations *p* < 0.05 corrected.

**Conclusion:** These findings suggest that the integrity of the contralesional CST may be used to track individual behavioral changes observed with BCI training after stroke.

## Introduction

Brain–computer interface (BCI) devices are being incorporated into new therapies for stroke rehabilitation. These devices translate neural activity into real-time feedback that can be used to train consistent brain activation patterns in association with specific mental states. BCI devices can be used safely and effectively to elicit functional gains in stroke survivors with persistent motor deficits (Broetz et al., 2010; Caria et al., 2011; Ramos-Murguialday et al., 2013; Ang et al., 2014; Mukaino et al., 2014; Young et al., 2014a; McCrimmon et al., 2015) and may enhance the efficacy of concurrent or associated therapies (Ramos-Murguialday et al., 2013; Ang et al., 2014; Mukaino et al., 2014), even after individuals reach a functional plateau using traditional therapies (Broetz et al., 2010; Caria et al., 2011; Mukaino et al., 2014).

The availability of real-time neural feedback allows the user to engage in reward-based neuromodulatory training, which is thought to induce use-dependent plasticity and facilitate functional recovery (Young et al., 2014d). Furthermore, the coupling of BCI devices with triggered functional electrical stimulation (FES) allows for resynchronization of cortical activation, peripheral activation, and sensory feedback. This strengthening of central-peripheral connections may then facilitate motor gains through Hebbian and other plasticity processes (Young et al., 2014d; McCrimmon et al., 2015).

There is some evidence that BCI therapies may effect behavioral gains through brain-related mechanisms. Changes in brain activation (Caria et al., 2011; Mukaino et al., 2014; Young et al., 2014c) and functional connectivity (Varkuti et al., 2013; Young et al., 2014b) have been observed in stroke patients receiving rehabilitative BCI therapies, and these changes have also been associated with behavioral gains (Caria et al., 2011; Varkuti et al., 2013; Mukaino et al., 2014; Young et al., 2014b,c). However, the mechanisms by which BCI training relates to observed changes in functional behaviors after stroke are poorly understood.

While a growing literature is examining functional brain changes with rehabilitative BCI therapies, far less has been documented in structural brain changes associated with their use. However, the structural integrity of white matter tracts is an important component in understanding motor impairment (Pineiro et al., 2000; Schiemanck et al., 2008), and changes in structural connectivity may be associated with functional reorganization after stroke (Li et al., 2015). Structural remodeling of these tracts has also been related to motor recovery (Liu et al., 2008; Schaechter et al., 2009) and is thought to be an adaptive, compensatory response after stroke (Schaechter et al., 2009). In particular, measures of corticospinal tract (CST) integrity have been associated with functional motor impairments in chronic stroke patients (Schaechter et al., 2009).

Diffusion tensor imaging (DTI) is a neuroimaging technique that allows for the quantitative assessment of white matter tract integrity after stroke (Werring et al., 2000; Thomalla et al., 2004; Stinear et al., 2007) and can be used to reconstruct white matter tracts in the brain (Mori and van Zijl, 2002; Stinear et al., 2007). DTI-derived measures of the CST can be considered valid structural surrogates of motor impairments after stroke and have been studied in relation to motor outcomes and motor recovery (Stinear et al., 2007; Schaechter et al., 2009; Fan et al., 2015).

Previous work examining white matter microstructure in stroke patients receiving BCI therapy have focused on the posterior limb of the internal capsule (PLIC; Song et al., 2014, 2015) or been limited case studies (Caria et al., 2011). These studies have identified correlations between DTI-derived metrics and motor performance (Song et al., 2014, 2015). Transcallosal motor fibers have also recently become the focus of studies on motor recovery after stroke (Lindenberg et al., 2012; Li et al., 2015), showing greater integrity in these fibers to be associated with better upper extremity motor function (Fan et al., 2015; Li et al., 2015) – potentially serving as predictors of motor improvement (Lindenberg et al., 2012). A better understanding of these types of brain–behavior relationships is critical in understanding the mechanism by which BCI training might mediate changes in motor performance and may also allow for the development of imaging biomarkers that can be used to track gains and response to this type of training in among stroke survivors with persistent motor impairments.

In contrast to previous work, this study presents an exploratory analysis of structural brain–behavior relationships in stroke survivors receiving BCI training using DTI-derived measures over the entire reconstructed CST. We examine changes in transcallosal motor fiber integrity for relationships with those in CST integrity and with individual gains. This study also examines how changes in the integrity of motor fibers derived from the CST relate to behavioral changes in motor function at the individual level both before and after the administration of BCI training. Given the neuromodulatory nature of BCI training and previous work suggesting activity-dependent neuroplasticity with this type of training (Young et al., 2014c), we hypothesize that BCI training will induce or modify structural brain–behavior relationships observed in a population of stroke patients with persistent upper extremity motor impairments.

## Materials and Methods

### Subject Recruitment

Subjects were recruited as part of an on-going stroke rehabilitation study investigating interventional BCI therapy targeting upper extremity motor function. This on-going study was registered with ClinicalTrials.gov (study ID NCT02098265). Inclusion criteria were: age 18 years or older; persistent upper extremity motor impairment resulting from stroke; and no other known neurologic, psychiatric, or developmental disabilities. Exclusion criteria were: allergy to electrode gel, surgical tape, and/or metals; concurrent treatment for infectious disease; apparent lesions or inflammation of the oral cavity; pregnancy or intention to become pregnant during the course of the study; and any contraindication for magnetic resonance imaging (MRI). This study was approved by the University of Wisconsin Health Sciences Institutional Review Board (Study ID 2015-0469); all subjects provided written informed consent upon enrollment. Detailed demographic information for each subject enrolled is provided in **Table 1**.

**Table 1:** Subject characteristics.

Subject	Sex	Age	Stroke lesion	Lesion duration	Handedness	Impaired hand	NIHSS	Group
1	M	52	L MCA	15	R	R	8	Experimental Only
2	M	68	L frontal lobe	3	R	R	0	Experimental Only
3	M	66	L MCA	23	R	R	6	Experimental Only
4	F	73	L MCA	2	A	R	0	Experimental Only
5	M	59	L MCA	28	R	R	2	Experimental Only
6	F	45	R MCA	99	R	L	6	Experimental Only
7	M	80	R occipital lobe	20	R	L	2	Experimental Only
8	M	76	R MCA	168	R	L	1	Experimental Only
9	M	50	R MCA	16	R	L	4	Experimental Only
10	F	75	R putamen	23	R	L	7	Crossover Control
11	M	61	L basal ganglia	17	R	R	0	Crossover Control
12	M	48	R pons	6	L	L	3	Crossover Control
13	M	48	R medulla	5	R	L	6	Crossover Control
14	M	77	R periventricular white matter, border of R motor cortex	22	L	L	1	Crossover Control
15	M	69	R PLIC, R putamen	90	R	L	1	Crossover Control
16	F	82	L PLIC, R parietal lobe, R pons	18	R	R	0	Crossover Control
17	F	64	R PLIC, R thalamus	3	R	L	1	Crossover Control
18	M	56	L MCA	11	R	R	2	Control Only
19	F	42	R MCA	87	R	L	3	Control Only

*M, male; F, female; L, left; R, right; MCA, middle cerebral artery; NIHSS, National Institutes of Health Stroke Scale. Ages are listed in years, lesion durations are listed in months since stroke. Handedness refers to pre-stroke handedness.*

### Study Schedule and Behavioral Measures

**FIGURE 1:**
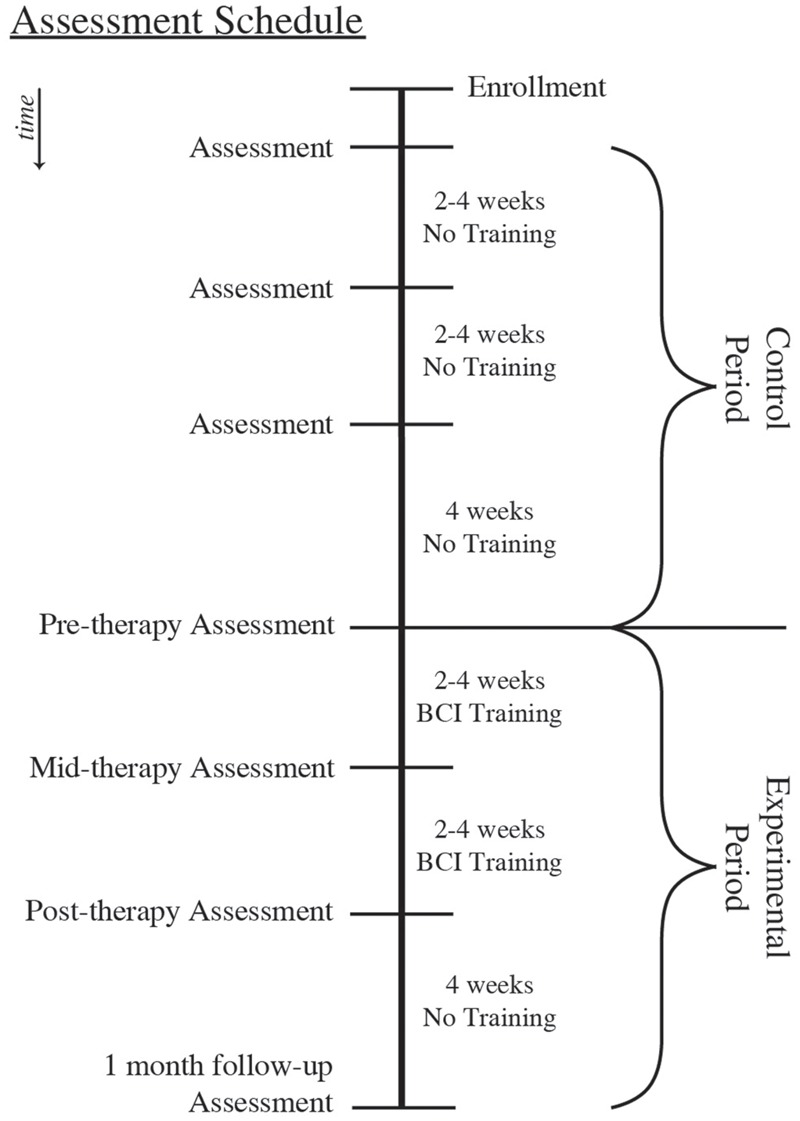
**Study Assessment Schedule.** BCI, Brain–Computer Interface.

This set of analyses uses a crossover-control design (**Figure 1**), with subjects randomly allocated to either the Crossover Control group or the Experimental Only group. Subjects assigned to the Crossover Control group complete seven assessment visits in addition to the period of BCI training, while those assigned to the Experimental Only group complete the period of BCI training along with only four assessments beginning with the pre-training assessment. This study design was chosen in order to balance limited resources with the need for data from a control period to determine whether any effects observed are attributable to the BCI training rather than practice effects. Behavioral measures and DTI scans were obtained on each assessment day. Between the pre-training and post-training assessments, subjects received at least 9 and up to 15 2-h sessions of BCI training.

The behavioral measures evaluated at each assessment included the Stroke Impact Scale (SIS; Carod-Artal et al., 2008), the Action Research Arm Test (ARAT; Lang et al., 2006), and the Nine-Hole Peg Test (9-HPT; Beebe and Lang, 2009). These measures were chosen in order to obtain both subjective (SIS) and objective (ARAT and 9-HPT) measures of upper extremity motor function. As this study focuses on upper extremity motor function, these analyses focus on the Hand Function domain of the SIS (SIS HF). SIS HF raw scores were transformed to reflect the percent possible points obtained by each subject. This study also analyzes total ARAT scores for the subject’s impaired hand and 9-HPT performance calculated as the average time (in seconds) needed to complete the task between two attempts both using the impaired hand.

### BCI Training Session Sequence

Brain–computer interface training procedures were consistent with those previously described (Song et al., 2014; Young et al., 2014a,b,c). Training sessions used a BCI system using BCI2000 software (Schalk et al., 2004) version 2 with in-house modifications for input from a 16-channel EEG cap and amplifier (Guger Technologies) and integration with tongue stimulation (TDU 01.30 Wicab, Inc.) and FES (LG-7500, LGMedSupply; Arduino 1.0.4). BCI training sessions used an open-loop screening task at the beginning of each session to determine optimal control signals as previously described (Wilson et al., 2009). Signals focused on the Mu (8–14 Hz) and Beta (18–26 Hz) frequency ranges detected by EEG over the motor cortex. Subjects were then guided through repeated trials of a closed-loop neurofeedback task in which attempted movements of each hand were cued using a visual cursor-and-target task with additional feedback provided in the form of tongue stimulation (Wilson et al., 2012) and triggered FES on the muscles of the impaired arm. Movements practiced during BCI training sessions involved repeated attempts at motion in the hand or wrist and varied among subjects based on subject preference and the baseline abilities and recovery goals for each individual.

### MRI Acquisition and Processing

Magnetic resonance imaging scans were obtained at each assessment on one of three 3 Tesla GE MR750 scanners equipped with high-speed gradients (Sigma GE Healthcare, Milwaukee, WI, USA) using an 8-channel head coil. Padding was used to help minimize head movement, and subjects were instructed to keep their head still during scans. Images were acquired using single-shot echo planar imaging with TR = 9000 ms, TE = 66.2 ms, flip angle = 90°, number of excitations = 1, field of view = 256 × 256, voxel size 1 mm × 1 mm × 2 mm, number of gradient encoded directions = 56, *b*-value = 1000 s/mm^2^. Each image comprised 75 axial plane slices of 2 mm thickness with no gap between slices. Slices were obtained using an interleaved acquisition to minimize cross-talk artifacts. Scans were reconstructed and preprocessed using FSL (v 5.0.7; Smith et al., 2004), correcting for motion and eddy current distortion and applying head-motion correction for gradient distortion using FSL’s eddy_correct function. Subsequent image processing was performed using 3D Slicer (v4.4.0^1^; Fedorov et al., 2012). Each scan was visually inspected by two researchers to identify and remove individual gradient images in which dropped signals or other artifacts compromised data quality (Stamm et al., 2015). Visual inspection of individual gradient images resulted in the deletion of one gradient direction from eight scans, the deletion of two gradient directions from eight scans, the deletion of three gradient directions from four scans, and the deletion of four gradient directions in two scans. After removing these gradient images, diffusion tensors were estimated for each voxel. ROI’s were manually drawn in native space on the DTI color map in the axial plane around the PLIC in each hemisphere, in the axial plane around each CST in the upper pons at the level of the superior cerebellar peduncle, and in the sagittal plane around the corpus callosum at the midline using a human white matter atlas for reference (Oishi et al., 2010). This approach was chosen to avoid registration issues that can arise when fitting data from individuals with large stroke lesions into a standard space.

Deterministic tractography was performed using 3D Slicer with a fractional anisotropy (FA) threshold of 0.15 (Mori and van Zijl, 2002; Stamm et al., 2015) with tracts originating from each PLIC ROI. Fibers were then restricted such that only fibers from a given PLIC which also passed through the ipsilateral CST region in the pons but did not cross the corpus callosum were retained. This process produced two distinct fiber bundles: the ipsilesional CST and the contralesional CST. Fibers traced from each PLIC that encountered both the ipsilateral CST region in the pons and the corpus callosum were used to represent transcallosal motor fibers. This method of tracing transcallosal motor fibers was employed because reliable reconstruction of these fibers between the primary motor cortices is known to be technically difficult when large cortical lesions or aging processes are involved, prompting the derivation of these fibers from the CST (Kwon et al., 2014). The Fiber Tract Scalar Measurements module in Slicer (v. 4.4.0-2015-07-15^1^; Fedorov et al., 2012) was used to obtain FA values along the length each of these three tracts (i.e., the ipsilesional CST, the contralesional CST, and the transcallosal motor fibers).

Fractional anisotropy is a normalized scalar index that quantifies the degree to which water is directionally restricted in its diffusion (Alexander et al., 2007) that can serve as a reliable marker of white matter microstructural status (Sidaros et al., 2008). FA measures along the CST have been shown to evolve (Schaechter et al., 2009; Lindenberg et al., 2012) and to relate to motor skill (Thomalla et al., 2004; Stinear et al., 2007; Schaechter et al., 2009) following stroke. Similarly, the asymmetry in FA values between the ipsilesional and contralesional CST’s, as reflected by asymmetric FA (aFA), has also been associated with motor function and motor recovery (Stinear et al., 2007), with greater asymmetries corresponding to poorer outcomes. FA values for the ipsilesional and contralesional CST’s were used to calculate aFA values using the formula aFA = (FA_contralesional_–FA_ipsilesional_)/(FA_contralesional_+FA_ipsilesional_) (Stinear et al., 2007; Schaechter et al., 2009; Song et al., 2014).

### Statistical Analysis

R statistical software (version 3.0.1) was used for all statistical analyses. A likelihood ratio test was used to evaluate linear mixed-effect models of FA values, aFA values, and behavioral scores to identify potential group by time interactions using data from the pre-training, mid-training, and post-training assessments along with data from the control period counterparts of these time points.

Changes in behavioral measures at each time point were normalized by calculating the percent change from baseline values. These percent change values were analyzed for correlations with changes in FA for each of the three fiber bundles of interest using generalized estimating equations (GEEs; Ballinger, 2004). In order to minimize the influence of floor and ceiling effects, subjects who consistently scored the absolute minimum or maximum on a given measure at all assessment points were excluded from correlation analyses using data from that outcome measure. Similarly, individuals from which no traceable fibers were obtained for a given fiber bundle were omitted from correlation analyses using data from that fiber bundle.

Thresholds for significance and trend toward significance were set *a priori* at *p* ≤ 0.05 and 0.05 < *p* < 0.1 respectively for all statistical analyses. All *p*-values were corrected for multiple comparisons using false discovery rate correction (Benjamini and Hochberg, 1995) before being compared to these thresholds.

## Results

### Participant Characteristics, Compliance, and Quality Control

Data was collected from 19 individuals. Subject characteristics are summarized in **Table 1**. Subjects comprised a predominantly right-handed cohort as determined using the Edinburgh Handedness Inventory (Oldfield, 1971), had an average age of 62.7 years (standard deviation 12.7 years), and were an average of 34.5 months (range: 2–168 months) post-stroke.

Among these subjects, nine completed the assessments around the period of BCI Training (**Table 1**, Experimental Only), eight completed both control and experimental period assessments (**Table 1**, Crossover Control), and two were randomly assigned to the Crossover Control group but had only completed the assessments scheduled prior to receiving any BCI Training at the time of analysis (**Table 1**, Control Only). One subject (Subject 11) did not complete the final follow-up assessment due to scheduling issues. DTI scans were not obtained for Subject 13 at the third Control assessment or for Subject 18 at the fourth MRI scan session due to time limitations while in the MRI scanner. A DTI scan was also not obtained for Subject 14 at the third Control assessment when the subject arrived on the wrong day and scanner time could not be rescheduled to accommodate this error. No adverse events were reported.

For one subject with lesions in both hemispheres (Subject 16), anatomical scans revealed that the lesion in the left PLIC was the only one to which the subject’s motor deficits were attributable, with remaining lesions relatively isolated from the corticomotor system. Thus, the left hemisphere was designated as ipsilesional and the right contralesional for further analysis.

### Neuroimaging and Behavioral Outcome Measures

**FIGURE 2:**
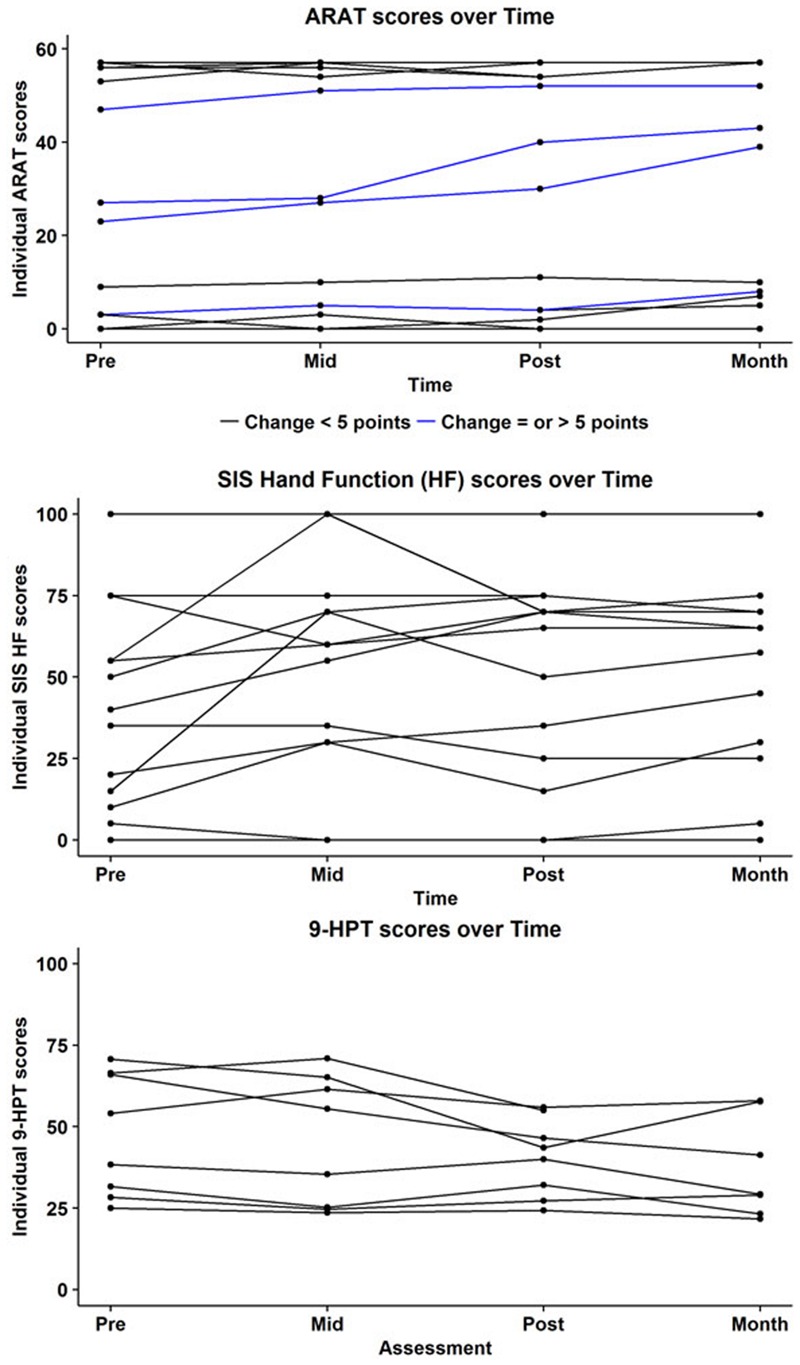
**Subject-wise behavioral scores at each time-point for the Action Research Arm Test (ARAT; **top**), Stroke Impact Scale (SIS; **middle**), and the Nine Hole Peg Test (9HPT; **bottom**).** Maximum score on the ARAT is 57. Blue lines indicate subjects who showed a positive change of atleast five points on the ARAT. Minimally Clinically Important Difference (MCID) in chronic stroke on this scale is 5.7 points. Subjects who were at floor level, i.e., (unable to perform) on the 9HPT are not represented here.

**FIGURE 3:**
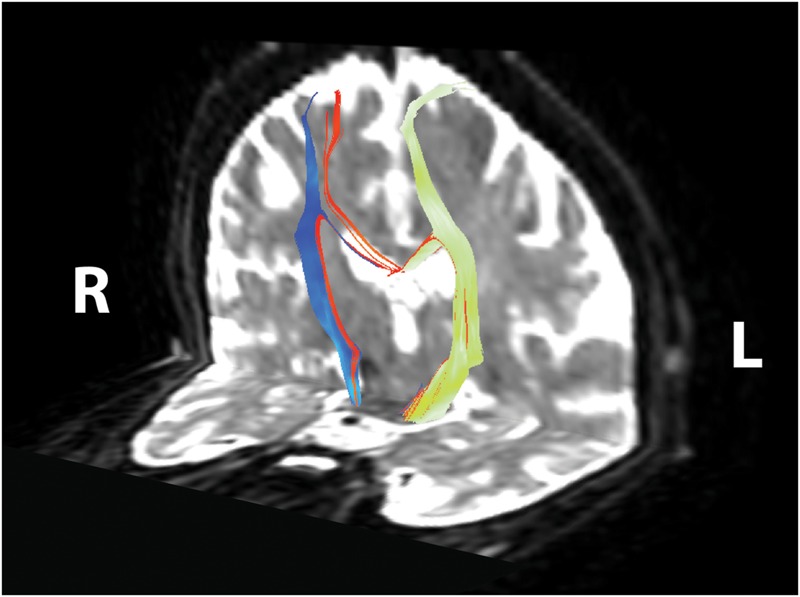
**Corticomotor fiber tracts.** Ipsilesional corticospinal tract shown in yellow–green, contralesional corticospinal tract shown in blue, transcallosal motor fibers shown in red. Reconstructed tracts overlaid on diffusion-weighted image in subject’s native space. R, right; L, left.

A complete set of behavioral scores and FA values for each subject is shown in **Table 2**. **Figure 2** shows behavioral scores for each subject at each time-point. Supplementary Table S1 shows the FA values for each subject at each time-point in the ipsilesional and contralesional CST.A representative tracing of each CST and the transcallosal motor fibers is presented in **Figure 3**. No significant group by time interactions were identified for any of the DTI-based metrics examined. Similarly, none of the behavioral measures examined were found to have any significant group by time interactions. There was one subject (Subject 19) for which no ipsilesional fibers could be traced at any time point. In eight subjects no traceable transcallosal fibers could be traced at any timepoint. Floor or ceiling effects were observed among seven subjects on the SIS HF, among six subjects on the ARAT, and among nine subjects on the 9-HPT.

**Table 2:** Assessment-specific behavioral scores.

Subj	Measure	Control 1	Control 2	Control 3	Pre- training	Mid- training	Post- training	1 Month
1	SIS HF				0	0	0	0
	ARAT				0	3	0	0
	9-HPT				Unable	Unable	Unable	Unable
2	SIS HF				40	55	70	75
	ARAT				57	57	57	57
	9-HPT				66	55.5	46.5	41.25
3	SIS HF				0	0	0	0
	ARAT				3	0	0	0
	9-HPT				Unable	Unable	Unable	Unable
4	SIS HF				50	70	50	57.5
	ARAT				56	56	54	57
	9-HPT				38.36	35.355	40	29.245
5	SIS HF				55	100	70	65
	ARAT				57	57	57	57
	9-HPT				31.67	25.25	32.145	23.27
6	SIS HF				0	0	0	0
	ARAT				3	5	4	5
	9-HPT				Unable	Unable	Unable	Unable
7	SIS HF				55	60	70	70
	ARAT				57	54	57	57
	9-HPT				54	61.5	55.955	58.01
8	SIS HF				0	0	0	5
	ARAT				9	10	11	10
	9-HPT				Unable	Unable	Unable	Unable
9	SIS HF				20	30	15	30
	ARAT				3	5	4	8
	9-HPT				Unable	Unable	Unable	Unable
10	SIS HF	0	0	0	5	0	0	0
	ARAT	0	0	0	0	0	0	0
	9-HPT	Unable	Unable	Unable	Unable	Unable	Unable	Unable
11	SIS HF	55	35	50	75	75	75	Not tested
	ARAT	54	57	51	53	57	54	
	9-HPT	106	76.5	74.5	66.485	71	55	
12	SIS HF	10	0	20	10	30	35	45
	ARAT	26	27	32	27	28	40	43
	9-HPT	Unable	Unable	Unable	Unable	Unable	Unable	Unable
13	SIS HF	0	0	0	0	0	0	0
	ARAT	0	0	0	3	0	2	7
	9-HPT	Unable	Unable	Unable	Unable	Unable	Unable	Unable
14	SIS HF	100	100	100	100	100	100	100
	ARAT	57	57	57	57	57	57	57
	9-HPT	26	30	27	28.29	24.705	27.215	29
15	SIS HF	30	40	25	35	35	25	25
	ARAT	17	25	31	23	27	30	39
	9-HPT	Unable	Unable	Unable	Unable	Unable	Unable	Unable
16	SIS HF	50	45	35	75	60	65	65
	ARAT	52	51	51	47	51	52	52
	9-HPT	48.935	49.825	56.5	70.76	65.16	43.575	57.7
17	SIS HF	40	30	45	15	70	75	70
	ARAT	56	55	NA	56	57	57	57
	9-HPT	35.705	27.5	29.545	24.97	23.57	24.31	21.715
18	SIS HF	30	45	45	55			
	ARAT	54	57	57	54			
	9-HPT	67	41.16	42	39.5			
19	SIS HF	10	0	25	0			
	ARAT	5	5	0	7			
	9-HPT	Unable	Unable	Unable	Unable			
Avg.	SIS HF	32.5	29.5	34.5	31.05263	40.2941	38.2352	37.9687
	ARAT	32.1	33.4	31	30.10526	30.8235	31.5294	31.625
	9-HPT	56.728	44.997	45.909	46.67056	45.255	40.5875	37.17
*SD*	SIS HF	30.8445	31.7498	28.9107	31.69205	36.2918	35.2642	34.3416
	ARAT	24.9597	24.9363	25.0898	25.1725	25.5177	25.6639	25.5652
	9-HPT	31.5442	19.75808	19.81304	17.86939	20.06641	11.97118	15.45992

*Stroke Impact Scale Scores presented as total transformed scores for the Hand Function domain. Action Research Arm Test scores presented as total points scored for the impaired upper extremity. Nine-Hole Peg Test times expressed in seconds. Subj, subject; SIS HF, Stroke Impact Scale Hand Function; ARAT, Action Research Arm Test; 9-HPT, Nine-Hole Peg Test; Avg., average; SD, standard deviation.*

### Relationships between Transcallosal FA and Other Corticospinal Tract DTI Metrics

**FIGURE 4:**
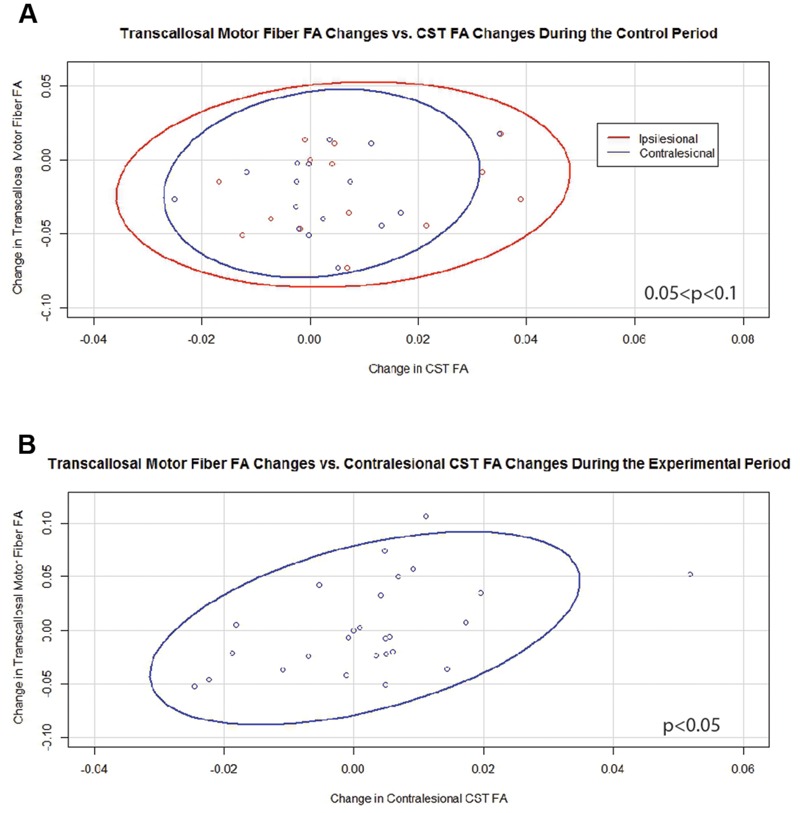
**Significant and trending correlations between changes in transcallosal motor fiber FA and changes in CST FA values. (A)** During the Control Period (0.05 < *p* < 0.1 for both relationships). **(B)** During the Experimental Period (*p* < 0.05). FA, fractional anisotropy; CST, corticospinal tract. Ovals represent data ellipses at the 95% confidence level.

Generalized estimating equation analysis showed that during the control period changes in transcallosal FA trended toward significance for relationships with changes in both ipsilesional and contralesional CST FA values (**Figure 4A**). In contrast, changes in transcallosal FA were positively correlated with changes in contralesional CST FA (**Figure 4B**) but not with changes in ipsilesional CST FA during the experimental period. There were no relationships identified between changes in transcallosal FA and changes in aFA throughout the study period.

### Correlations between Brain and Behavioral Measures

**FIGURE 5:**
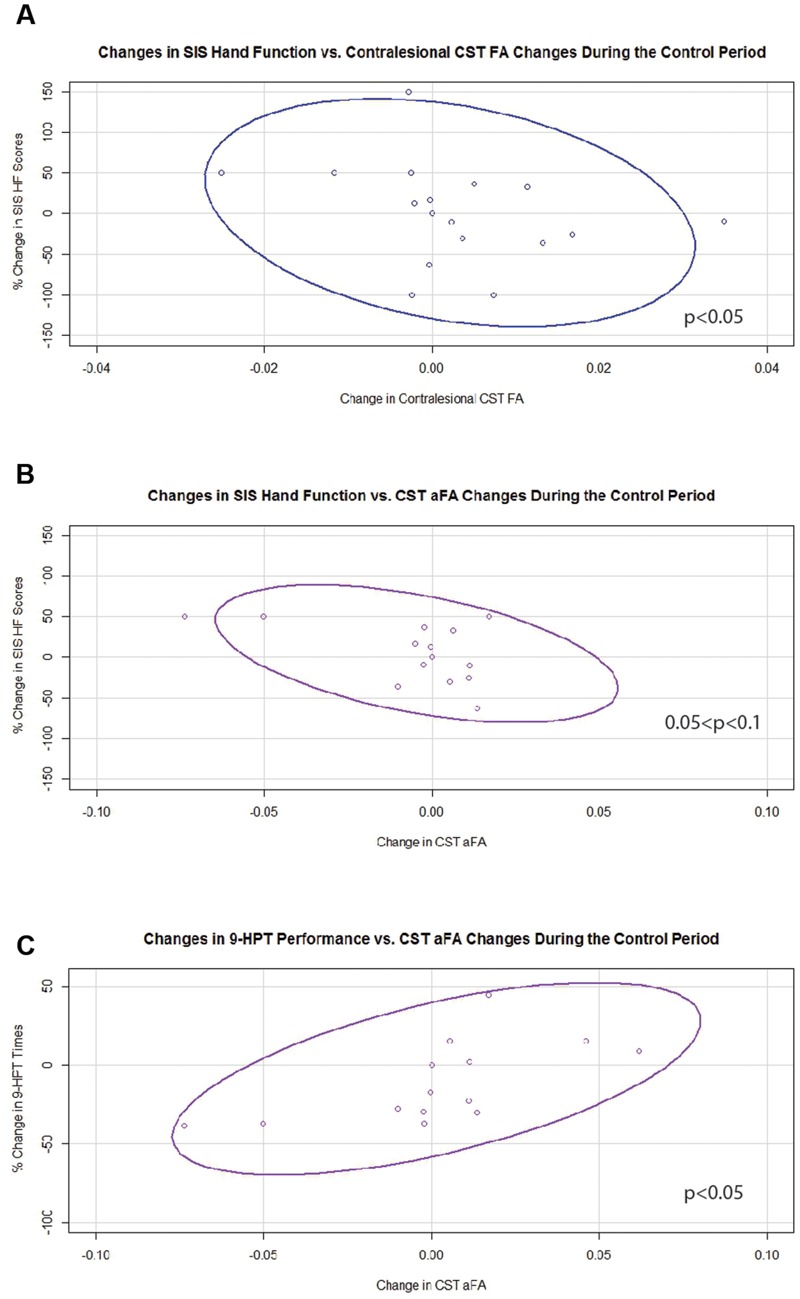
**Significant and trending correlations between CST FA or aFA changes and behavioral changes during the Control Period. (A)** Relationship between changes in contralesional CST FA and changes in SIS Hand Function Scores (*p* < 0.05). **(B)** Relationship between changes in aFA and changes in SIS Hand Function Scores (0.05 < *p* < 0.1). **(C)** Relationship between changes in aFA and changes in 9-HPT performance (*p* < 0.05). CST, corticospinal tract; FA, fractional anisotropy; aFA, asymmetric fractional anisotropy; SIS, Stroke Impact Scale; 9-HPT, Nine-Hole Peg Test. Ovals represent data ellipses at the 95% confidence level.

Generalized estimating equation analysis showed that during the control period, changes in contralesional CST FA significantly and negatively correlated with changes in SIS HF scores (**Figure 5A**). Changes in aFA during this period correlated positively with changes in 9-HPT times (**Figure 5C**) and trended toward significance for a negative correlation with changes in SIS HF scores (**Figure 5B**). No relationships were identified between changes in ipsilesional CST FA or transcallosal FA with changes in behavior during the control period.

**FIGURE 6:**
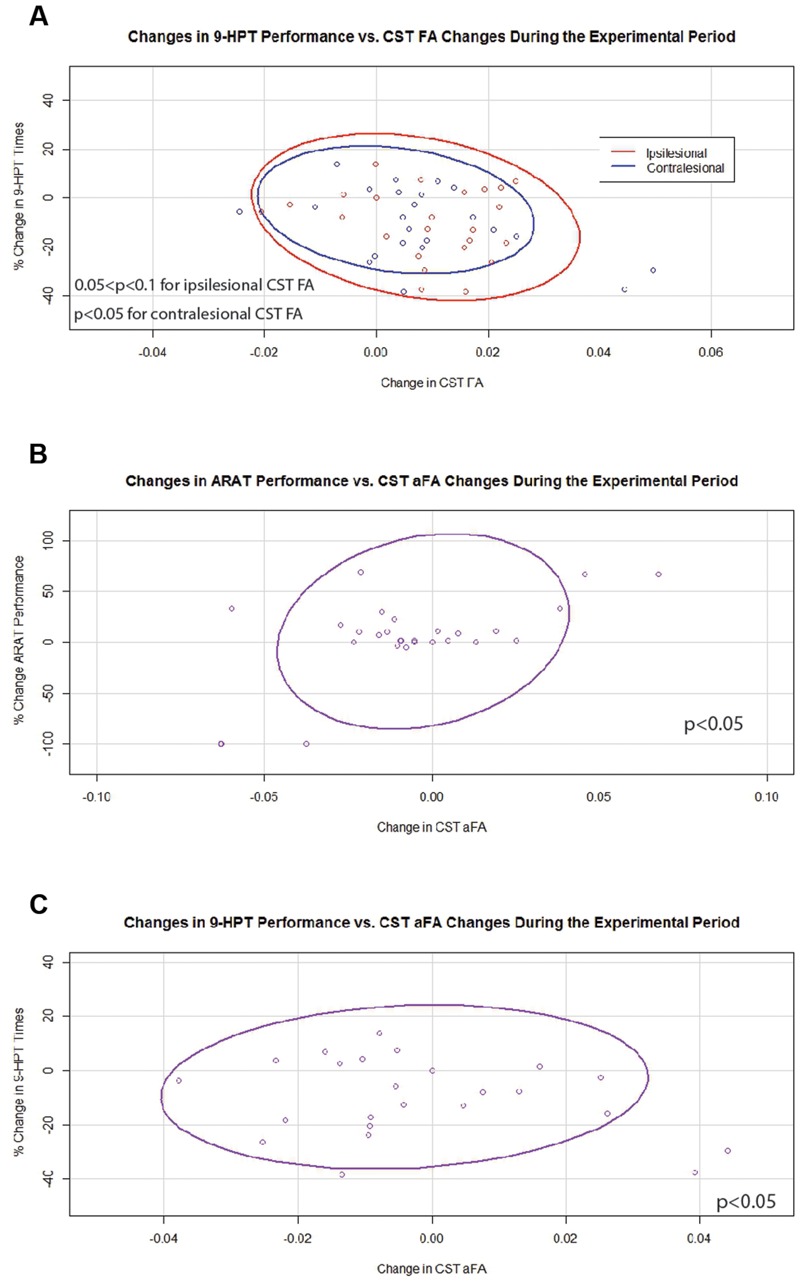
**Significant and trending correlations between CST FA or aFA and behavioral changes during the Experimental Period. (A)** Relationships between changes in CST FA and changes in 9-HPT performance (*p* < 0.05 for contralesional CST FA changes; 0.05 < *p* < 0.1 for ipsilesional CST FA changes). **(B)** Relationship between changes in aFA and changes in ARAT scores (*p* < 0.05). **(C)** Relationship between changes in aFA and changes in 9-HPT performance (*p* < 0.05). CST, corticospinal tract; FA, fractional anisotropy; aFA, asymmetric fractional anisotropy; 9-HPT, Nine-Hole Peg Test; ARAT, Action Research Arm Test. Ovals represent data ellipses at the 95% confidence level. Data ellipses were calculated excluding outlier points, but all individual data points including outliers are shown in each graph.

In contrast, during the experimental period changes in 9-HPT times were found to correlate negatively with changes in FA values that were significant for contralesional CST FA and trended toward significance for ipsilesional CST FA (**Figure 6A**). Changes in aFA were also significantly and positively correlated with changes in ARAT scores and with changes in 9-HPT times (**Figures 6B,C**). No relationships were identified between changes in transcallosal FA and changes in behavior during the experimental period.

## Discussion

Although preliminary in nature, this study provides one of the first examinations of relationships among ipsilesional, contralesional, and transcallosal motor fiber integrity, behavioral changes in recovering stroke patients, and how these relationships change with the administration of BCI training. Although no group by time interactions were identified, changes in transcallosal FA measurements correlated with changes in contralesional CST FA during the experimental period with BCI training. Similarly, individual changes in CST FA and aFA correlated with those in behavioral measures in a pattern that suggests an increasingly compensatory role for the contralesional CST with BCI training. Previous work examining relationships between DTI metrics and motor performance in stroke patients receiving BCI therapy has shown greater structural integrity in the ipsilesional PLIC to be associated with better function (Song et al., 2014, 2015).

Studies have suggested that structural remodeling of white matter tracts occurs following stroke and can play a role in motor recovery (Schaechter et al., 2009; Lindenberg et al., 2012; Fan et al., 2015). This remodeling is often described as an initial decrease in FA after stroke followed by a subsequent increase in FA associated with white matter reorganization, angiogenesis, and enhanced neural connectivity (Jiang et al., 2006; Wang et al., 2006; Ding et al., 2008; van der Zijden et al., 2008). However, not all studies have been able to replicate the finding of altered FA values following stroke, particularly in the contralesional CST (Lindenberg et al., 2012).

A previous examination of stroke patients receiving BCI therapy found that DTI-derived metrics from the PLIC were not significantly different across time (Song et al., 2015). This is consistent with our analysis, which failed to identify group by time interactions in DTI-derived metrics. While these metrics did not change significantly at the group level, individual variations in these measures were found to interrelate and correlated with individual changes in behavioral measures.

Transcallosal fibers tracked in this study include inter-hemispheric connections that play an important role in recovery after stroke (Perez and Cohen, 2009). Previous studies have shown an association between the integrity of transcallosal fibers and motor performance following stroke (Lindenberg et al., 2012; Fan et al., 2015). We derived transcallosal fibers from the CST (similar to Kwon et al., 2014) rather than directly from cortical motor regions primarily due to a large number of our patients having lesions in the primary motor and premotor regions. Changes in transcallosal FA trended toward significant correlations with changes in both ipsilesional and contralesional CST FA values during the control period. These findings are consistent with previous work by Lindenberg et al. (2012), who identified reductions in both transcallosal and ipsilesional CST FA in chronic stroke patients. The significant relationship identified between the integrity of transcallosal motor fibers and that of fibers in the contralesional CST with the administration of BCI training, along with the observation that individual changes in transcallosal motor fibers no longer track with those in the ipsilesional CST, suggest an increased coupling between transcallosal and contralesional motor fiber integrity associated with BCI training. Animal experiments have shown sprouting of contralesional axons that form connections with denervated nuclei after stroke in a manner enhanced by post-stroke treatments associated with improved recovery (Lee et al., 2004; Ramic et al., 2006; Andrews et al., 2008; Liu et al., 2008). It may be that processes such as these, involving axonal remodeling of both contralesional and transcallosal motor fibers, are stimulated by training with a BCI system.

While no significant differences were observed at the group level, individual changes in white matter integrity tracked with variations in behavioral measures both before and after subjects received BCI training. Such relationships are important in further understanding the mechanisms that underlie motor recovery after stroke, as some studies have shown regional FA values to correlate not with baseline performance but with individual changes in motor impairment observed with therapy (Stinear et al., 2007; Lindenberg et al., 2012).

During the control period, increased FA of the contralesional CST was associated with reduced SIS HF scores. Similarly, increased aFA was associated with poorer 9-HPT performance and trended toward significance with reduced SIS HF scores. Together, these findings suggest that in the absence of BCI training a structural imbalance favoring the contralesional CST is associated with worse motor function across the spectrum of deficit severity. Previous work has shown greater damage to white matter tracts and larger cortical lesions to be associated with poorer motor function and greater bilateral recruitment after stroke (Miyai et al., 2001; Wang et al., 2012). Another study has also shown greater output through homologous tracts in the contralesional CST in stroke patients with less integrity in the ipsilesional CST (Cunningham et al., 2015).

The persistent imbalance in FA between the contralesional and ipsilesional CST has been called characteristic of chronic white matter degeneration due to loss of structural integrity (Werring et al., 2000; Stinear et al., 2007; Lindenberg et al., 2012). These asymmetries in CST FA can be strongly predictive of clinical performance and functional potential in more severely impaired stroke patients but not so in those with less direct damage to the corticospinal system (Stinear et al., 2007). A differential pattern in CST integrity between stroke patients with high and low severity in motor impairment was also observed when bilateral elevations in CST FA were observed in patients with more preserved motor function, while patients with more severe motor deficits had significant reductions in CST FA (Schaechter et al., 2009).

The role of contralesional areas in motor recovery remains incompletely understood (Johansen-Berg et al., 2002; Lindenberg et al., 2012; Young et al., 2014c); evidence grows for both compensatory (Johansen-Berg et al., 2002; Liu et al., 2008; Schaechter et al., 2009; Young et al., 2014c) and maladaptive (Fregni and Pascual-Leone, 2006; Murphy and Corbett, 2009) contributions while the determinants of the relative strengths of these conflicting forces remains unknown (Schaechter et al., 2009). Significant asymmetries in CST FA may also reflect a maladaptive disruption to the balance of interhemispheric inhibition between the ipsilesional and contralesional sides of the motor system (Stinear et al., 2007). Given this context, our findings may reflect the need for increased compensatory recruitment of the contralesional hemisphere in subjects with more damage to the ipsilesional CST or may reflect a maladaptive process in those with less damage to the ipsilesional CST and less severe motor deficits.

As initially hypothesized, a number of relationships between individual fluctuations in FA and those in behavioral measures appeared to change with BCI training. During the experimental period, increases in FA for both the ipsilesional and contralesional CST’s were significantly associated or trended toward significant association with improvements in 9-HPT performance. However, increases in aFA continued to be associated with worse 9-HPT performance. These findings suggest that while individual improvements in the integrity of each CST may signify beneficial neuroplastic or compensatory changes in stroke patients with more mild impairments (i.e., those able to perform the 9-HPT with no floor effects), a pattern of improvement skewed to the contralesional CST may continue to represent a maladaptive process. This finding is consistent with other work that has shown stroke patients with better motor skill to have bilateral increases in CST FA (Schaechter et al., 2009). However, one study found aFA predictive of functional improvements only in stroke patients with more significant ipsilesional CST damage (Stinear et al., 2007). It may be that these differences are attributable to differences in stroke populations studied or in the type of therapy paradigm (i.e., BCI vs. non-BCI) examined.

Changes in aFA toward increased dominance of the contralesional CST were associated with improvements in ARAT scores during the experimental period, suggesting that individual improvements in motor function with BCI training achieved by stroke patients with more significant motor impairments (i.e., those with no ceiling effects on ARAT) may be effected by a neuroplastic or compensatory process that relies more heavily on the contralesional CST. That this relationship and the significant correlation between changes in transcallosal motor fiber integrity and changes in contralesional CST FA were only observed during the experimental period highlights the importance of the contralesional CST in stroke patients with motor impairments receiving BCI training. It has been suggested that neuromodulatory interventions may enhance compensatory activity in the contralesional hemisphere after stroke (Stinear et al., 2007; Young et al., 2014c). Previous work has also suggested an important role for the contralesional hemisphere in a similar cohort of stroke patients receiving BCI therapy (Young et al., 2014c). These findings further support the idea that the neuromodulatory nature of BCI training may induce structural and functional changes in the contralesional hemisphere that can be related to individual motor gains.

Interestingly, no associations were identified between individual changes in ipsilesional CST FA with changes in behavior during the control period, nor were any associations identified between changes in transcallosal motor fiber integrity and those in behavioral measures. Transcallosal motor fibers are known to play a role in interhemispheric inhibition between the primary motor cortices (Lindenberg et al., 2012; Wang et al., 2012; Kwon et al., 2014). Models of pathological imbalance in these inter-hemispheric interactions after stroke have highlighted the importance of transcallosal motor fibers in recovery (Jang et al., 2009; Perez and Cohen, 2009; Carter et al., 2012). However, the exact role of these connections in stroke recovery remains poorly understood, as imbalances in CST integrity and output have not been found to relate to transcallosal inhibition in chronic stroke (Cunningham et al., 2015).

Our findings are somewhat consistent with work that found a significant relationship between transcallosal motor fibers FA and baseline motor performance but less so between these FA values and individual motor gains (Lindenberg et al., 2012). In contrast, another study of stroke patients receiving bilateral robotic training found those with greater FA increases in transcallosal and ipsilesional motor tracts over the course of therapy showed greater gains in motor function (Fan et al., 2015). Differences between these findings and those of the present study may be due to a number of factors, including differences in methodology used to identify transcallosal motor fibers and in therapy modalities. Deriving transcallosal fibers from the CST (Kwon et al., 2014) rather than directly from cortical motor regions (Lindenberg et al., 2012; Fan et al., 2015) likely results in different subpopulations of transcallosal connections analyzed among studies. The nature of the BCI system used, which simultaneously trains neuromodulatory efforts in both hemispheres, is also different from the transcranial direct current stimulation and bimanual robotic therapies used in the previous works mentioned. These differences may also affect whether or not changes in transcallosal motor fibers relate to individual motor gains.

Some limitations should be acknowledged when considering the findings presented. This cohort represents a heterogeneous range of stroke lesions and motor deficit severities. This heterogeneity may have contributed to the lack of group by time interactions across the control and experimental periods. With different subpopulations of stroke patients showing different responses and patterns of neuroplastic change associated with recovery (Stinear et al., 2007; Schaechter et al., 2009; Lindenberg et al., 2012), it may be that differential effects of the BCI training examined in this study on subgroups within the cohort are masked and may not reach statistical significance when the cohort is analyzed as a whole. However, limited sample size precludes meaningful subanalysis of the current cohort among those with mild, moderate, and severe deficits at this time.

In this study we did not explicitly test predictors of BCI induced recovery. However, in previous work, we have already shown that DTI-FA values can be used to track changes and to predict recovery in stroke patients, and have found that the more intact the PLIC [a small area of the motor tract (CST)] is in a stroke patient, the better their recovery (Song et al., 2015). Another limitation of the current study is that while this study identified a number of relationships among individual FA changes and behavioral changes, it remains limited in its ability to ascribe these changes to specific cellular and molecular processes. Numerous factors have been shown to influence FA, including myelination, axonal characteristics, and fiber density (Beaulieu, 2002; Assaf and Pasternak, 2008), with damage to both axons and myelin known to result from stroke. Imaging limitations may also impact the conclusions relating to FA. For example, the current technique cannot distinguish between fibers in the CST and alternate corticospinal fibers, which are known to traverse through the PLIC but have not been fully mapped in human anatomical studies (Schmahmann et al., 2004; Lindenberg et al., 2010). Changes in alternate and corticofugal fibers may also relate to changes in motor skill (Schaechter et al., 2009; Lindenberg et al., 2010) and may mask FA changes in the true CST (Lindenberg et al., 2010). Similarly, the current technique cannot distinguish among subpopulations of motor fibers within the CST specific to different parts of the homunculus (e.g., face vs. hand vs. foot). The development of more advanced imaging techniques that are able to reliably distinguish among these types of motor fiber subpopulations in individuals with stroke lesions or other neuropathologies would be of great benefit to future studies of the relationships between behavioral outcomes and motor fiber structural integrity.

Future studies comparing BCI-based approaches with other modalities will allow for a better understanding of mechanisms by which different components of BCI and non-BCI interventions elicit motor gains. While the pattern of brain–behavioral relationships changed between the control and experimental periods, we cannot disentangle the relative contributions of each component of the BCI system used. Future work incorporating larger sample sizes and more homogeneous cohorts will allow for a better understanding of differential responses to BCI training among various stroke subpopulations and the mechanisms by which these responses are effected.

Despite the limitations of these preliminary analyses, individual changes in white matter integrity of the CST tracked with those in transcallosal fibers and with changes in behavioral outcome measures. Changes in these patterns of association between the two study periods suggest that an imbalance favoring the contralesional hemisphere prior to BCI training may represent a maladaptive process during stroke recovery, but that with BCI training the contralesional hemisphere may serve a compensatory role in facilitating behavioral gains. Such a pattern may inform the future development of imaging biomarkers intended to track individual response in stroke patients receiving training on a BCI system like the one used in this study. Further investigation into the differential roles of each hemisphere in recovery among stroke patients receiving BCI therapies will guide the design and application of future rehabilitative therapies that incorporate neuromodulatory training.

## Author Contributions

BY assisted in subject recruitment, data collection, data analysis, and writing. JMS assisted with image processing and quality control. JS assisted with subject recruitment, data collection, and data processing. AR assisted with data collection. VN assisted with subject recruitment, data collection, data analysis, and writing. MT provided TDU hardware and expertise. DE assisted with study design and data analysis. KC assisted with subject recruitment. JAS assisted with study design, subject recruitment, and manuscript editing. JW is one of two lead PI’s on this project and supervised the technical and engineering aspects of the work. VP is one of two lead PI’s on this project and supervised the neuroimaging and neuroscience aspects of this work. In addition to these respective contributions to the concept, design, data acquisition, and interpretation of data for this project, all authors were also involved in critically revising the work for intellectual content and approve of the final submission.

## Conflict of Interest Statement

There is one patent pending on the closed-loop neurofeedback device used for the training administered in this study (Pending U.S. Patent Application No. 12/715,090). This patent was filed jointly by the two lead investigators JW and VP. Otherwise, the authors have no conflicts of interest to report, as this research was conducted in the absence of commercial and financial relationships that might compromise the integrity of the results reported herein.

## Footnotes

1 http://www.slicer.org
