# Supplementary material for: Brain–Computer Interface Training after Stroke Affects Patterns of Brain–Behavior Relationships in Corticospinal Motor Fibers
Source: Front Hum Neurosci. 2016 Sep 16;10:457. doi: 10.3389/fnhum.2016.00457 (PMC5025476; doi:10.3389/fnhum.2016.00457)
Supplement: Supplementary file 1 [file Table_1.DOCX]

Supplemental Table S1. Assessment-Specific Fractional Anisotropy Values. Subj = Subject; Ipsi = Ipsilesional; Contra = Contralesional; CST = Corticospinal tract; Trans = Transcallosal; NF = No surviving fibers traced; Avg. = Average; SD = Standard Deviation.

| **Subj** | **Measure** | **Control 1** | **Control 2** | **Control 3** | **Pre- Training** | **Mid- Training** | **Post- Training** | **1 Month** |
| --- | --- | --- | --- | --- | --- | --- | --- | --- |
| **1** | **Ipsi CST** |  | | | 0.444831 | 0.439642 | 0.419243 | 0.473845 |
|  | **Contra CST** |  |  |  | 0.462354 | 0.461463 | 0.514168 | 0.481947 |
|  | **Trans** |  |  |  | 0.467123 | 0.460552 | 0.519296 | 0.50222 |
| **2** | **Ipsi CST** |  | | | 0.409312 | 0.411081 | 0.417979 | 0.417427 |
|  | **Contra CST** |  |  |  | 0.429271 | 0.454304 | 0.4789 | 0.473637 |
|  | **Trans** |  |  |  | 0.406202 | NF | NF | NF |
| **3** | **Ipsi CST** |  | | | 0.37117 | 0.400042 | 0.416931 | 0.423232 |
|  | **Contra CST** |  |  |  | 0.446704 | 0.446422 | 0.442036 | 0.448945 |
|  | **Trans** |  |  |  | NF | 0.430745 | NF | 0.454785 |
| **4** | **Ipsi CST** |  | | | 0.405772 | 0.399724 | 0.42804 | 0.413283 |
|  | **Contra CST** |  |  |  | 0.421051 | 0.425757 | 0.434992 | 0.420802 |
|  | **Trans** |  |  |  | 0.346356 | 0.420417 | NF | NF |
| **5** | **Ipsi CST** |  | | | 0.42057 | 0.436372 | 0.414762 | 0.441192 |
|  | **Contra CST** |  |  |  | 0.443409 | 0.451599 | 0.451563 | 0.442224 |
|  | **Trans** |  |  |  | NF | 0.37486 | NF | NF |
| **6** | **Ipsi CST** |  | | | 0.323388 | 0.316967 | 0.305499 | NF |
|  | **Contra CST** |  |  |  | 0.443898 | 0.478125 | 0.453719 | 0.428763 |
|  | **Trans** |  |  |  | NF | NF | NF | NF |
| **7** | **Ipsi CST** |  | | | 0.431179 | 0.431119 | 0.450486 | 0.439063 |
|  | **Contra CST** |  |  |  | 0.447323 | 0.440281 | 0.446102 | 0.450796 |
|  | **Trans** |  |  |  | 0.481912 | 0.45763 | 0.440032 | 0.458634 |
| **8** | **Ipsi CST** |  | | | 0.507278 | 0.486864 | 0.487885 | 0.46656 |
|  | **Contra CST** |  |  |  | 0.503719 | 0.485039 | 0.473735 | 0.481376 |
|  | **Trans** |  |  |  | 0.490924 | 0.469841 | NF | 0.445337 |
| **9** | **Ipsi CST** |  | | | 0.377035 | 0.332433 | 0.425845 | 0.391537 |
|  | **Contra CST** |  |  |  | 0.438759 | 0.443667 | 0.439633 | 0.444239 |
|  | **Trans** |  |  |  | 0.422892 | 0.401144 | 0.425368 | 0.417299 |
| **10** | **Ipsi CST** | 0.378456 | 0.402351 | 0.388572 | 0.395596 | 0.410515 | 0.393634 | 0.403068 |
|  | **Contra CST** | 0.479883 | 0.478007 | 0.472143 | 0.489208 | 0.458512 | 0.479242 | 0.458954 |
|  | **Trans** | NF | 0.449498 | NF | NF | NF | NF | NF |
| **11** | **Ipsi CST** | 0.42827 | 0.449662 | 0.463437 | 0.435081 | 0.459964 | 0.451839 | Not Scanned |
|  | **Contra CST** | 0.455136 | 0.468335 | 0.490005 | 0.460217 | 0.471254 | 0.469321 |  |
|  | **Trans** | 0.472159 | 0.428077 | 0.490372 | 0.399123 | 0.505854 | 0.456621 |  |
| **12** | **Ipsi CST** | NF | 0.428711 | 0.408631 | NF | NF | 0.427551 | 0.412365 |
|  | **Contra CST** | NF | 0.470998 | 0.467198 | NF | 0.478446 | 0.468752 | 0.469984 |
|  | **Trans** | NF | NF | 0.416358 | NF | NF | NF | NF |
| **13** | **Ipsi CST** | 0.403517 | 0.409957 | NF | NF | 0.380492 | NF | NF |
|  | **Contra CST** | 0.460273 | 0.465162 | 0.444675 | 0.453782 | 0.451384 | 0.436582 | 0.440973 |
|  | **Trans** | NF | NF | NF | NF | NF | NF | NF |
| **14** | **Ipsi CST** | 0.45779 | 0.435297 | Not Scanned | 0.420875 | 0.430138 | 0.442833 | 0.436656 |
|  | **Contra CST** | 0.429983 | 0.448221 |  | 0.447357 | 0.453289 | 0.436386 | 0.451469 |
|  | **Trans** | NF | 0.434616 |  | 0.447502 | 0.427816 | 0.410994 | 0.480428 |
| **15** | **Ipsi CST** | 0.420072 | 0.424456 | Not Scanned | 0.424158 | 0.431295 | 0.44994 | 0.437673 |
|  | **Contra CST** | 0.487922 | 0.499248 |  | 0.487677 | 0.469489 | 0.501983 | 0.482283 |
|  | **Trans** | 0.501194 | 0.512323 |  | 0.498966 | 0.504181 | 0.463128 | 0.541641 |
| **16** | **Ipsi CST** | 0.431963 | 0.42473 | 0.43092 | 0.415167 | 0.425118 | 0.431221 | 0.438461 |
|  | **Contra CST** | 0.437294 | 0.439685 | 0.440938 | 0.434784 | 0.452053 | 0.439653 | 0.439555 |
|  | **Trans** | 0.427359 | 0.387417 | 0.441241 | 0.412509 | 0.420294 | 0.405481 | 0.361835 |
| **17** | **Ipsi CST** | 0.460927 | 0.468056 | 0.459127 | 0.448388 | 0.427811 | 0.432992 | 0.465635 |
|  | **Contra CST** | 0.437626 | 0.454334 | 0.435532 | 0.437317 | 0.412784 | 0.444143 | 0.458399 |
|  | **Trans** | 0.498437 | 0.462982 | 0.451969 | 0.44728 | 0.394887 | 0.497798 | NF |
| **18** | **Ipsi CST** | 0.409482 | 0.448312 | 0.441274 | Not Scanned |  | | |
|  | **Contra CST** | 0.454591 | 0.42947 | 0.442949 |  |  |  |  |
|  | **Trans** | 0.400134 | 0.373931 | 0.391972 |  |  |  |  |
| **19** | **Ipsi CST** | NF | NF | NF | NF |  | | |
|  | **Contra CST** | 0.449872 | 0.447472 | 0.447157 | 0.45722 |  |  |  |
|  | **Trans** | 0.428443 | 0.426332 | 0.396676 | 0.414007 |  |  |  |
| **Avg.** | **Ipsi CST** | 0.42381 | 0.432392 | 0.431994 | 0.41532 | 0.413724 | 0.424793 | 0.432857 |
|  | **Contra CST** | 0.454731 | 0.460093 | 0.455075 | 0.453179 | 0.454933 | 0.459465 | 0.454647 |
|  | **Trans** | 0.454621 | 0.434397 | 0.431431 | 0.436233 | 0.439018 | 0.45234 | 0.457772 |
| **SD** | **Ipsi CST** | 0.027534 | 0.020534 | 0.029132 | 0.041042 | 0.042705 | 0.038209 | 0.024488 |
|  | **Contra CST** | 0.019358 | 0.020424 | 0.019116 | 0.022201 | 0.018721 | 0.023992 | 0.018978 |
|  | **Trans** | 0.041938 | 0.043237 | 0.037374 | 0.044526 | 0.041409 | 0.040477 | 0.054155 |
